# MATERNAL DIFFERENTIAL TREATMENT AND ADULT CHILDREN’S DEPRESSIVE SYMPTOMS IN THE FACE OF COGNITIVE IMPAIRMENT

**DOI:** 10.1093/geroni/igad104.0054

**Published:** 2023-12-21

**Authors:** J Jill Suitor, Megan Gilligan, Robert Frase, Destiny Ogle, Ranran He

**Affiliations:** Purdue University, West Lafayette, Indiana, United States; University of Missouri, Columbia, Missouri, United States; Southern Illinois University, Carbondale, Illinois, United States; Purdue University, West Lafayette, Indiana, United States; Sociology, West Lafayette, Indiana, United States

## Abstract

Parental differential treatment has been shown to have a negative impact on adult children’s psychological well-being, particularly among daughters. In this paper, we explore whether mothers’ cognitive impairment (CI) moderates the impact of maternal favoritism/disfavoritism on psychological well-being when children are entering their later years (M=60), and their mothers are in their 80s-90s. We address these questions using mixed-methods data from 297 adult children who participated in wave 3 of the Within-Family Differences Study. Quantitative and qualitative analyses revealed that the effects of perceived favoritism/ disfavoritism were shaped by the combination of child’s gender and mothers’ CI status. Perceptions of disfavoritism, particularly regarding mothers’ disappointment, affected daughters’ depressive symptoms; however, the effect was decidedly muted by mothers’ CI. In the case of mothers’ preferences regarding their future caregivers, the differences by cognitive status were particularly striking. Contrary to expectations, daughters’ perceptions that they were their mothers’ preferred caregivers predicted depressive symptoms when mothers showed no signs of CI, but had no impact when mothers were cognitively impaired. Among sons, depressive symptoms were not affected by mothers’ favoritism or disfavoritism, regardless of mothers’ CI status; a gender difference in the impact of mothers’ appraisals on well-being that we have seen emerge across the 20 years we have followed these families. Qualitative analyses suggest that mothers’ CI reduced daughters’ emotional reactions to their mothers’ differential treatment because they believed that their mothers had less control of their behaviors and less awareness of the negative impact of their appraisals and expectations.

